# The intratumoral microbiota in breast cancer: from basic research to clinical translation

**DOI:** 10.1080/19490976.2025.2560695

**Published:** 2025-09-15

**Authors:** Yan Xu, Meng-Chuan Wang

**Affiliations:** a Department of Pharmacy, The Affiliated People’s Hospital of Ningbo University, Ningbo, China; b Department of Pharmacy, Affiliated Cixi Hospital, Wenzhou Medical University, Ningbo, China

**Keywords:** Tumor microbiome, cancer biomarkers, breast cancer, immunotherapy, gut microbiota

## Abstract

Breast cancer remains a leading global malignancy among women, with increasing incidence and mortality. Recent advances in multi-omics technologies have revealed the presence of diverse microbial communities in the tumor microenvironment, comprising bacteria, viruses, and fungi. These microbes play complex roles within tumor initiation and progression, affecting local inflammation and modulating host metabolism, genomic stability, and immune responses. Emerging evidence indicates that the intratumoral microbiota holds diagnostic potential and represents a novel biomarker for molecular subtyping and prognosis. Furthermore, intratumoral microbiota offer new avenues for targeted interventions, such as engineered bacteria and phage therapy, which may overcome limitations of conventional treatments. This review summarizes current insights into the composition, colonization pathways, mechanisms, and clinical applications of intratumoral microbiota, underscoring their potential to advance precision medicine in breast cancer.

## 1. Introduction

Breast cancer is the most prevalent malignant tumor among women worldwide, with persistently high incidence and mortality. Currently, over 2.4 million new cases are diagnosed annually, making it the leading cause of cancer-related death in women.^1^ Despite advances in early screening and comprehensive treatment, the prognosis for advanced or metastatic breast cancer remains poor. Early-stage breast cancer often presents asymptomatically, contributing to delayed diagnosis and elevated mortality. Triple-negative breast cancer (TNBC) exemplifies this challenge, with a 5-year survival rate below 50%.^2^ Advanced breast cancer is difficult to cure by surgery alone, and the efficacy of chemotherapy, radiotherapy, and immunotherapy still faces many challenges.^3^ Therefore, the diagnosis and treatment of breast cancer still need to be further explored.

Breast cancer is a complex disease influenced by genetic, hormonal, and environmental factors.^4^ In recent years, emerging evidence indicates that the human microbiome plays a significant role in the occurrence and progression of breast cancer.^5^ The microbiome encompasses the diverse microbial communities, including bacteria, archaea, viruses, fungi, and parasites that coexist within the host.^6^ Although the gut is the main habitat of the human microbiome, these microorganisms also widely exist in other parts, such as skin, mouth, respiratory tract, genitourinary tract, and breast tissue.^7^ These microorganisms are not only involved in maintaining normal physiological functions, but also related to the occurrence of a variety of diseases, including cancer. Contrary to the historical view of breast tissue as sterile, recent studies confirm distinct microbial communities within both healthy and malignant breast tissue, with significant compositional shifts in tumors.^8^ Intratumoral microbiota and their metabolites may affect the biological characteristics, immune response, and therapeutic effect of breast tumors. For example, *Fusobacterium nucleates* has been proven to be carcinogenic in a variety of digestive system tumors, and its role in breast cancer has been gradually revealed.^9^ Moreover, the diversity of intratumoral microbiota is closely associated with different breast cancer subtypes, and the presence of specific microorganisms is linked to the prognosis of patients.^10^

This review summarizes the current research progress of intratumoral microbiota in breast cancer, focusing on its potential role in tumourigenesis, as biomarkers, and therapeutic applications in breast cancer. We also discussed the challenges and future directions for translating intratumoral microbiota research into clinical practice for breast cancer. Understanding the complex relationship between intratumoral microbiota and breast cancer may lead to novel diagnostic tools, prognostic markers, and targeted therapies to improve patient outcomes.

## 2. Characteristics and genesis of intratumoral microbiota in breast cancer

### 2.1. Characteristics of intratumoral microbiota in breast cancer

**Figure 1.**
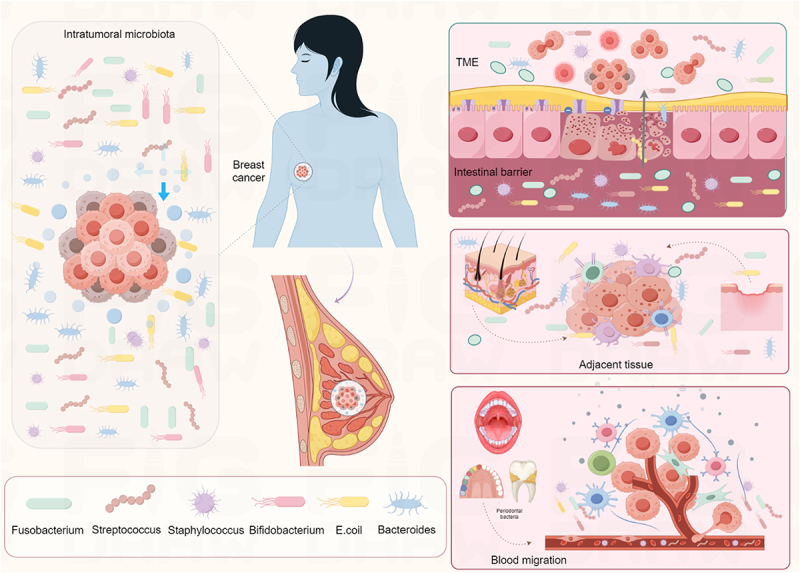
Characteristics and genesis of intratumoral microbiota in breast cancer. A variety of microbial communities have been identified in the breast cancer microenvironment, including *Staphylococcus*, *Streptococcus*, *Fusobacterium*, *Bifidobacterium*, *Bacteroidetes*, etc. The potential genesis of intratumoral microbiota can be classified into the following three main ways:^1^ impaired intestinal mucosal barrier function. The gut microbiota translocated through the intestinal mammary axis.^2^ adjacent barrier structures. If the integrity of the skin or nipple areola complex is destroyed, the local microbiota will directly invade.^3^ blood circulation. Periodontal pathogens spread through bacteremia and colonize breast tissue through blood migration. Made by Figdraw.

Microorganisms are widespread in human tissues, particularly in the gut, which harbors approximately 3 × 10^13^ microorganisms.^11^ Previous studies have shown that changes in the composition of the gut microbiota community are closely related to the occurrence and progression of breast cancer.^12,13^ Advances in high-throughput sequencing have progressively uncovered the presence and potential roles of microbes in breast cancer tissues. The microbial ecosystem in breast cancer showed complex and dynamic characteristics. In recent years, through the application of new-generation sequencing (NGS) and other molecular technologies, a diverse array of bacteria, fungi, and viruses has been identified in breast cancer tissues, showing distinct tissue specificity and molecular subtype dependence.^14–16^ The main bacterial phyla of microorganisms in breast cancer include *Firmicutes*, *Proteobacteria*, *Actinobacteria*, and *Bacteroidetes*, with *Proteobacteria* being the most dominant.^17–20^ In breast cancer tissues, the abundance of *Staphylococcus*, *Streptococcus*, *Fusobacterium*, *and Bifidobacterium* was significantly higher than that in adjacent normal tissues.^20^ Specifically, *Staphylococcus* and *Streptococcus* are highly abundant in tumor tissues, whereas *Bacteroidetes* and *Fusobacterium* have lower relative abundance. *Anaerococcus*, which is central in benign breast tissues, is absent in tumor tissues.^21,22^ In addition, fungal microorganisms such as *Candida* and *Aspergillus* have also been detected in breast cancer tissues (Figure 1).

#### 2.1.1. Subtype-specific microbial signatures in breast cancer

Breast cancer can be classified by the expression levels of hormone receptor (HR), including estrogen receptor (ER) and progesterone receptor (PR), as well as human epidermal growth factor receptor 2 (HER2) in the cancer cells.^2^ Breast cancer can be classified into subgroups HR-positive (HR+), HER2-positive (HER2+), TNBC (HR-, HER2-), and triple-positive breast cancer (TPBC) (HR+, HER2+).^2,3^ Emerging evidence indicates that the intratumoral microbiota composition varies notably among these subtypes, suggesting subtype-specific microbial signatures that may influence tumor biology, progression, and therapeutic response.^17^ *Fusobacterium nucleatum* is significantly enriched in TNBC, while *Staphylococcus epidermidis* is more abundant in HR+ breast cancer, and *Actinomyces* is enriched in HER2+ breast cancer.^23^ Elevated levels of *Bacillus*, *Mucor*, *Nodaviridae*, *Toxocara*, and *Trichophyton* in TNBC samples are correlated with better prognosis.^23^ Banerjee et al. have also found that the microbial diversity in HR+ samples is notably higher compared to that of TNBC, which exhibited the lowest microbial diversity.^24^ In HR+ samples, genera such as *Klebsiella*, *Stenotrophomonas*, and *Neodiprostomum* displayed elevated abundance and were significantly correlated with prolonged disease-free survival (DFS). Further analysis of TPBC revealed that high levels of *Orientia*, *Klebsiella*, *Fusobacterium*, *Azorhizobium*, *Yersinia*, *Arthroderma*, *Anelloviridae*, *Angiostrongylus*, and *Toxocara* correlate significantly with reduced DFS or OS.^24^ Human papillomavirus (HPV) and Epstein-Barr virus (EBV) DNA/RNA have been detected variably across breast cancer subtypes, with some evidence suggesting a higher prevalence in TNBC and HR+ cancers. While the exact oncogenic roles of these viruses in breast cancer remain under investigation, their presence highlights potential viral contributions to subtype-specific tumorigenesis (Table 1).^24^

**Table 1.** Characterization of the intratumoral microbiota in breast cancer.

Microbiome compositions	Quantitative dynamics	Clinical samples	Specific description	Ref
*Fusobacterium, Atopobium, Gluconacetobacter, Hydrogenophaga Lactobacillus*	Increase	33 breast cancer tissue samples	The microbiota of breast tissue is different from that of breast skin tissue, breast skin swabs, and oral swabs, and is related to the enrichment of low-abundance groups	^15^
*Bacteroidetes, Firmicutes, Proteobacteria, Actinobacteria*	Increase	36 normal breast tissue samples, 10 breast cancer samples	There are significant differences in microbial composition diversity between tumor tissue and normal tissue	^16^
*Ralstonia picketii*	Decrease	44 breast tissue samples, including benign, malignant, and adjacent breast tissue samples	The relative abundance of *Ralstonia picketii* decreases in malignant tumors, while the genus Pseudomonas significantly increases in breast tissue.	^17^
*Actinobacteria*, *Proteobacteria*	Increase Decrease	34 breast tumoral and the adjacent non-tumoral tissue samples	The levels of Actinobacteria and Proteobacteria were, respectively, higher and lower in tumor tissues.	^19^
*Acinetobacter, Rhodobacter, Micrococcus, Corynebacteriales, Priestia megaterium, Streptomyces*, *Siphoviridea, Myoviridae,*	Increase	23 breast tumors and normal tissues	*Streptomyces, Siphavirida, and Myoviridae* are enriched in Chinese patients, while other microorganisms are enriched in Slovak patients.	^20^
*Bacillaceae, Burkholderiaceae, Corynebacteriaceae, Streptococcaceae, Staphylococcaceae*	Increase	49 normal breast tissues, 15 prediagnostic tissues, 49 afterdiagnostic adjacent, 46 tumor tissues	Early events of breast cancer development may be related to cancer susceptibility.	^21^
*Propionibacterium Staphylococcus*	Decrease	221 breast cancer patients, 18 individuals predisposed to breast cancer, and 69 controls	Showed negative associations with oncogenic immune features.	^22^
*Fusobacterium nucleatum*, *Staphylococcus epidermidis*, *Actinomyces Bacillus, Mucor, Nodaviridae, Toxocara, Trichophyton*		50 HR+, 34 HER2+, 24 TPBC, 40 TNBC, and 20 breast control samples	Intratumoral microbiota composition varied notably among subtypes of breast cancer.	^23^
*Klebsiella*, *Stenotrophomonas, Neodiprostomum*, *Orientia, Klebsiella, Fusobacterium, Azorhizobium, Yersinia, Arthroderma, Anelloviridae, Angiostrongylus, Toxocara, HPV, EBV*	Increase	95–105 formalin-fixed paraffin-embedded samples for each subtype, 20 matched control samples	Correlations between the presence and absence of specific microbes in breast cancer subtypes with the clinical outcomes have been proven.	^24^
*Methylobacterium radiotolerans*	Increase	paired normal and breast cancer tissues	The total bacterial DNA load in tumors decreases. Bacterial DNA load is negatively correlated with advanced diseases	^25^
*Listeria spp*. *Haemophilus. influenza*	Increase	TCGA data	Listeria is associated with genes related to epithelial-mesenchymal transition. *Haemophilus influenzae* may exist in the surrounding matrix material and be related to the proliferation pathway.	^26^

#### 2.1.2. Spatial heterogeneity of intratumoral microbiota in breast cancer

Breast cancer intratumoral microbiota exhibit significant spatial heterogeneity. Different tumor regions may harbor distinct microbial communities, which can influence local tumor biology and treatment responses. Studies have found that the density of *Fusobacterium nucleatum* in the front of tumor invasion is higher than that in the center of the tumor. The presence of *Proteus* is significantly increased in tumor tissues, while the abundance of *Actinomyces* is higher in non-cancerous adjacent tissues. *Haemophilus influenzae* may exist in the surrounding matrix materials and is significantly related to the proliferation pathway.^25,26^ Spatial metabonomic studies have revealed that the concentration of lactic acid in areas enriched with *Candida albicans* increases 2.4-fold, potentially mediating local immunosuppression. This spatial heterogeneity may increase the complexity of breast cancer progression and lead to treatment resistance.^27^

#### 2.1.3. Temporal dynamics of intratumoral microbiota in breast cancer

The intratumoral microbiota is not static but evolves during breast cancer progression. The microbial composition of early-stage breast cancer may differ from that of advanced or metastatic cancers. Research has shown that as tumor size and grade increase, the relative abundance of *Bacteroidaceae* decreases while that of *Agrococcus* increases.^28^ Moreover, tumor microorganisms may respond to therapeutic interventions such as chemotherapy or surgery. Understanding the temporal dynamics of intratumoral microbiota is crucial for developing personalized treatment strategies that can adapt to the changes in intratumoral microbiota.

#### 2.1.4. Intratumoral microbiota vary by race and sex in breast cancer patients

The intratumoral microbiota also vary according to the race and gender of breast cancer patients. Studies have found that *Pseudomonas* and *Methylobacter* are present in Asian female tumors, and *Amycolatopsis* in black female tumors.^29,30^ Notably, breast tissues from non-Hispanic Black women have a higher abundance of the genus *Ralstonia* compared to non-Hispanic White (NHW) tumors.^31^ Differences in the microbiome of male and female mammary glands have also been reported. *Tenericutes*, especially *Mycoplasma* and *Mycobacterium*, are associated with the occurrence of breast cancer in both sexes.^32^ In women, dysbacteriosis spreads throughout the breast tissue, while in men, it is more localized to the tumor site.

### 2.2. Genesis of intratumoral microbiota in breast cancer

The detection of microorganisms within tumors employs techniques such as fluorescence in situ hybridization (FISH), polymerase chain reaction (PCR), and 16S rRNA gene sequencing.^33^ The tumor microenvironment harbors microorganisms originating from diverse sources, which are influenced by tumor-specific factors like hypoxia, immunosuppression, and vascular abnormalities that facilitate microbial colonization and proliferation. The potential origins of these microorganisms are primarily categorized into three types (Figure 1). Firstly, destruction of mucosal barrier. There are abundant microorganisms in the mucosal organs of the human body, such as the lungs, gastrointestinal tract, and skin. Infection, trauma, dietary factors, and genetic mutations can all lead to the breakdown of the body’s mucosal barrier.^34^ Normally, mucosal barriers prevent microorganisms from penetrating deep tissues, but tumors can compromise their integrity, increasing the risk of microbial invasion.^35^ The gut is widely considered to be the main source of intratumoral microbiota. Studies have confirmed that intestinal bacteria such as *Fusobacterium nucleatum* can migrate to breast tissue through the blood or local, and their colonization and reproduction within tumor tissues have also been confirmed.^36,37^ Additionally, oral microorganisms represent a significant potential source. The oral cavity relates to the respiratory tract, and microorganisms in the oral cavity can migrate to the breast tissue through bacteremia or swallowing.^38^ In a study on a canine breast tumor model, *Bacteroides* could migrate from the oral cavity to the gut and ultimately establish in distant breast tumors.^39,40^

Secondly, adjacent tissues are the pathway of microbial invasion. Organs typically considered sterile, like the mammary gland, are connected to external environments via ductal systems. Under conditions of immunosuppression and hypoxia, local microorganisms can colonize these tissues.^7^ For example, skin-associated bacteria such as *Staphylococcus epidermidis* and *Micrococcus luteus* have been identified in breast tumors, suggesting they may enter the breast duct through the nipple and spread via lobules and ducts.^41^ The nipple-areolar complex’s resident bacteria, like *Staphylococcus*, can ascend retrogradely through the ductal system, proliferating within breast tissue. Clinical studies report a higher detection rate of *Staphylococcus* in nipple-areolar secretions from breast cancer patients compared to healthy controls.^42^

Thirdly, blood circulation is a crucial route for microorganisms to enter the tumor microenvironment. Microorganisms can break through the blood-brain barrier and intestinal barrier through the vascular system, enter the blood circulation, and colonize in breast tissue. Transient bacteremia during periodontal disease can promote the invasion of oral *Fusobacterium nucleatum* into the blood, leading to its translocation to the breast. Specifically, *Fusobacterium nucleatum* is transferred to breast tissue through the lacteal intestinal axis, direct nipple contact, and blood transmission. Subsequently, Fusobacterium autotransporter protein 2 (Fap2) was used to anchor the Fusobacterium adhesin A and lipopolysaccharide to the tumor tissue, and the virulence factors Fusobacterium adhesin A and lipopolysaccharide were used to promote the proliferation.^43^ In mice fed a high-fat diet, the colonization of *Escherichia coli* (*E. coli*) in breast tissue increased 8-fold. Intestinal microbial metabolites such as short-chain fatty acids (SCFAs) can also affect the immune microenvironment of breast tissue with blood flow and regulate the occurrence and development of tumors^44^ (Figure 1).

In conclusion, the sources of intratumoral microbiota are diverse and regulated by numerous factors. Their dynamic changes hold great significance for the biological and clinical aspects of breast cancer. In the future, in-depth studies on the source and migration mechanism of microorganisms will help to develop intervention strategies for microorganisms and improve the diagnosis and treatment of breast cancer.

### 2.3. Interactions between intratumoral and gut microbiota in breast cancer

**Figure 2.**
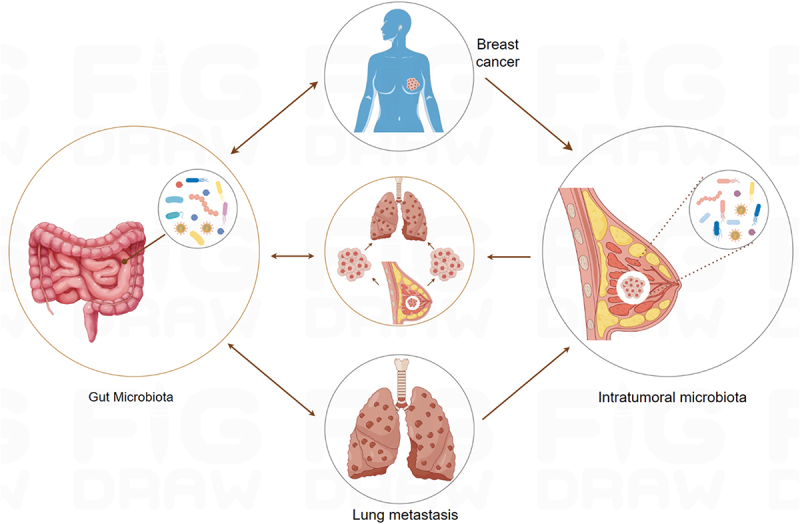
The interaction between intratumoral and gut microbiota in breast cancer. Gut dysbacteriosis can lead to the impairment of intestinal barrier function, making bacteria and metabolites enter the systemic circulation and then invade breast tissue, forming a vicious cycle of “gut-tumor-microbiota” interaction. Microbiota can promote the migration of microorganisms and metabolites by destroying the intestinal vascular barrier, forming a pre-metastatic microenvironment, and promoting lung metastasis of breast cancer. Made by Figdraw.

As the largest microbial community in the human body, the role of gut microbiota in the occurrence, development, and prognosis of breast cancer has been confirmed.^45^ There are complex and dynamic interactions between intratumoral and gut microbiota in breast cancer. This cross-organ microecological association is often conceptualized as “gut-mammary axis,” which plays an important role in tumor development and treatment response (Figure 2).

From the perspective of biological mechanisms, the imbalance of gut microorganisms (dysbacteriosis) may lead to the impairment of intestinal barrier function, making bacteria and their metabolites enter systemic circulation, and then affect breast tissue.^45,46^ Moreover, systemic inflammatory responses triggered by gut microbiota imbalance can alter the breast tissue microenvironment, favoring microbial growth and colonization within tumors.^11^ Gut microbiota can also affect the tumor microenvironment through metabolite-mediated signal transduction.^47^ For example, SCFAs, such as butyrate, produced by the metabolism of gut microbiota can reach the breast tissue through blood circulation, and indirectly shape the immunosuppressive or pro-inflammatory state in tumors by regulating the activity of immune cells (Treg cells, T cells).^47^ Conversely, substances like lipopolysaccharides released by specific tumor-resident microorganisms (*Staphylococcus* and *Streptococcus*) can be transported through the bloodstream to the intestine, damaging the integrity of the intestinal barrier, inducing gut dysbiosis, and establishing a vicious cycle of “gut-tumor” microecological interactions.^43^ Additionally, these microbial communities exert a synergistic influence on breast cancer treatment outcomes. The composition and structure of gut microbiota can affect the efficacy and toxicity of chemotherapy drugs such as docetaxel by regulating the activity of host drug metabolic enzymes (Cytochrome P450), while intratumoral microbiota directly change the local drug concentration through their own metabolic functions (degradation of drug molecules), both contributing to treatment resistance.^48^

In addition, the oral cavity and intestines, as the main habitats of microorganisms in the human body, are closely connected through the digestive tract, and fecal oral transmission is an important mechanism for their connection.^49^ Normally, most oral microorganisms are inactivated by gastric acid, but when the oral microbiota is imbalanced or gastric acid secretion is reduced, some oral pathogenic bacteria may colonize abnormally in the intestine, disrupt the balance of gut microbiota, alter the composition and function of the microbiota, weaken the intestinal barrier, and accelerate inflammation.^50^ Research on canine breast tumor models also shows that *Bacteroides* can migrate from the oral cavity to the intestine and ultimately colonize breast tumors.^39^ Oral microbiota can directly affect the breast tumor microenvironment. *Fusobacterium nucleatum* is a typical oral anaerobic bacterium commonly found in colorectal cancer and associated with poor prognosis.^51^ Research has found that *Fusobacterium nucleatum* can migrate to breast tissue through bacteremia or swallowing, and colonize mouse breast tumors, then indirectly affects systemic inflammation and immune status through microbial invasion and secretion of the toxic factor FadA adhesin.^35,38^ Moreover, studies have shown that oral microbial diversity is correlated with the prognosis of breast cancer, and the presence of periodontal pathogens may increase the risk of breast cancer. The FadA adhesin carried by periodontal bacteria can promote the invasion of breast epithelial cells.^40^ This indicates that the oral, gut, and tumor microbiota are not isolated ecosystems, but communicate with each other through complex networks and jointly participate in the regulation of host health.

Therefore, an in-depth study of the relationship between gut and intratumoral microbiota will help to reveal the potential mechanism of breast cancer occurrence and development, and provide new strategies and targets for early diagnosis, treatment, and prevention of breast cancer.

## 3. Mechanism of intratumoral microbiota in breast cancer

### 3.1. The role of intratumoral microbiota in breast cancer tumorigenesis

**Figure 3.**
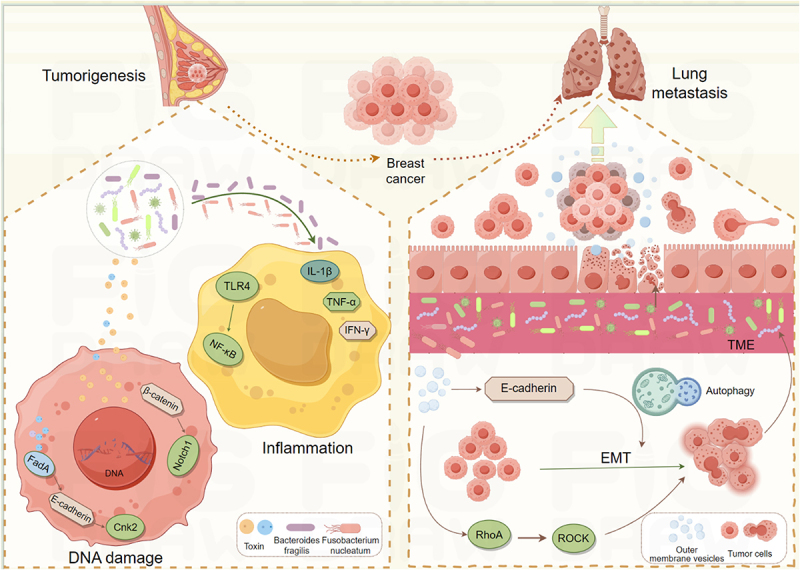
Mechanism of intratumoral microbiota in breast cancer. Intratumoral microbiota can stimulate the expression of Th1 cell-mediated inflammatory response and induce chronic inflammation by regulating cytokines such as IL-1β, TNF-α, and IFN-γ, thus driving tumorigenesis. Toxins produced by microorganisms can cause DNA double-strand breaks. *Fusobacterium nucleatum* secreted adhesin FadA to activate the E-cadherin/β-catenin pathway, upregulate CHK2 level, and induce DNA damage. Intratumoral microbiota can reach distant organs such as the lung by destroying the intestinal vascular barrier and blood diffusion, providing a physical basis for transfer and forming a pre-transfer microenvironment. In addition, microorganisms can affect cell signaling pathways and cell morphology, promote epithelial-mesenchymal transition (EMT), and thus promote the invasion and migration of cancer cells. Made by Figdraw.

The International Agency for Research on Cancer has identified 11 microorganisms with direct carcinogenic potential, including *Helicobacter pylori*, HPV, hepatitis B virus (HBV), and hepatitis C virus (HCV). These pathogens are responsible for approximately 1.9 million new cancer cases annually.^52^ Intratumoral microbiota can promote breast cancer development through various mechanisms, such as inducing inflammation, modulating immunity, and causing DNA damage (Figure 3).

#### 3.1.1. Inflammation and immunity

The microbiota can induce chronic inflammation by regulating the expression of cytokines, which can drive tumorigenesis. Dysbacteriosis, such as the increase of harmful bacteria such as *E. coli* and *Fusobacterium nucleatum*, can destroy the mucosal barrier, release pro-inflammatory factors and cancer mediators, promote persistent inflammatory response, and increase the risk of cancer.^53^ Microorganisms in the tumor microenvironment can reshape the local immune state in a variety of ways and promote the occurrence of breast cancer.^3^ For example, *Helicobacter pylori* can indirectly affect the carcinogenesis of the gastrointestinal tract and other tissues by recruiting neutrophils and monocytes, releasing interleukin-1β (IL-1β), tumor necrosis factor-α (TNF-α), and interferon-γ (IFN-γ), and stimulating a Th1 cell-mediated inflammatory response.^54^ Recent studies have revealed that *Fusobacterium nucleatum* promotes the formation of an immunosuppressive microenvironment by activating the TLR4/NF-κB signaling pathway. In the breast tumor microenvironment, the proportion of myeloid suppressor cells (MDSCs) and regulatory T cells (Tregs) increases, inhibiting anti-tumor immune responses.^54^ In addition, microbial metabolites such as butyrate have immunomodulatory effects, though their impact depends on concentration and microenvironment conditions.^55^

#### 3.1.2. DNA damage

Toxins produced by microorganisms, such as colibactin, can cause DNA double-strand breaks, promote gene mutation, and genomic instability. The study found that strains carrying the clbb gene were closely related to BRCA1/2 mutation patients, suggesting a potential role of microorganisms in genome damage.^56^ Guo et al. found that *Fusobacterium nucleatum*, which is abundant in the human breast cancer microbiome, secretes adhesin FadA, which activates the E-cadherin/β-catenin pathway, upregulates CHK2 levels, and induces DNA damage.^57^ Additionally, enterotoxigenic Bacteroides fragilis (ETBF), which colonizes the gut, increases spermine oxidase expression in breast tissue, leading to reactive oxygen species (ROS) production and γ-H2A activation, thereby causing DNA damage.^58^ Viral infections are also significant. Recent evidence has shown that HPV is detected in some breast cancer samples, particularly in young patients. The HPV E6/E7 proteins may promote cell proliferation and DNA damage by inhibiting the p53 and Rb pathways.^59^

### 3.2. The role of intratumoral microbiota in breast cancer metastasis

The process of cancer metastasis is complex, which is regulated by the intrinsic characteristics of tumor cells and the microenvironment. Although the 5-year relative survival rate of breast cancer is about 70%, the prognosis of patients with metastatic breast cancer is significantly poorer. Intratumoral microbiota can promote the process of metastasis and help cancer cells cope with a variety of physical, chemical, and biological barriers.^60^

#### 3.2.1. Pre-metastatic niche formation and metastasis promotion

Microorganisms can promote the migration of microorganisms and their metabolites by disrupting the intestinal vascular barrier, forming a pre-metastatic microenvironment. Intratumoral microbiota spread through the blood and reach distant organs such as the liver, providing a physical basis for metastasis.^61^ In the spontaneous breast tumor mouse model (MMTV PyMT), bacteria associated with circulating tumor cells regulate the RhoA/Rock signaling pathway. This interaction alters the actin cytoskeleton, enhances host cell resistance to fluid shear stress, and promotes breast cancer lung metastasis.^61^ Similarly, outer membrane vesicles from *Fusobacterium nucleatum* alter EMT-related protein levels and activate intracellular autophagy pathways, thereby promoting lung metastasis in tumor-bearing mice.^62^ Small extracellular vesicles derived from *Fusobacterium nucleatum* also drive tumor growth and metastasis through TLR4 signaling.^63^

#### 3.2.2. Microbial influence on cell signaling and microenvironment

Certain microorganisms can affect cell signaling pathways and morphology, promoting epithelial-mesenchymal transition (EMT) and enhancing cancer cell invasion and migration. Studies have identified shared microbial characteristics between normal adjacent tissues and tumor tissues in breast cancer patients. These microorganisms interact with the host and influence local recurrence risk.^64,65^ Microorganisms also modulate the tumor microenvironment via metabolites and signaling factors, regulating tumor cell behavior and stromal cell interactions. For example, SCFAs like butyrate exhibit anti-inflammatory and anti-tumor properties but can also promote tumor cell invasion.^66^ In contrast, hydrogen sulfide produced by some anaerobic bacteria enhances tumor cell proliferation and migration. Elevated lactic acid levels in breast cancer tissues not only fuel tumor cells but also inhibit cytotoxic T lymphocyte (CTL) and natural killer (NK) cell function, creating an immunosuppressive microenvironment.^67^ Malassezia fungal enrichment increases local lactic acid concentrations, suppressing anti-tumor immune cell activity and fulfilling tumor energy requirements. *Candida albicans* produces lactic acid through glycolysis, activating the GPR81 receptor and stimulating vascular endothelial growth factor (VEGF) secretion, thereby promoting angiogenesis. Animal studies show that antifungal agents fluconazole and itraconazole significantly reduce tumor metastasis, highlighting *Candida albicans*’ potential role in breast cancer metastasis.^68,69^ Some bacteria express β-glucuronidase, which hydrolyzes estradiol glycoside conjugates to release free estradiol, promoting ER+ breast cancer growth.^70^ This mechanism offers new insights into hormone-dependent breast cancer pathogenesis and suggests therapeutic potential in targeting microbial metabolic pathways.

In summary, intratumoral microbiota play a crucial role in breast cancer metastasis through multiple mechanisms, including pre-metastatic niche formation, cell signaling modulation, and microenvironment alteration. Understanding these interactions can pave the way for developing novel therapeutic strategies to combat metastatic breast cancer.

### 3.3. The role of intratumoral microbiota in drug resistance of breast tumor cells

Chemoresistance remains a major obstacle in effective breast cancer treatment, often leading to tumor recurrence and poor clinical outcomes. Increasing evidence indicates that the intratumoral microbiota plays a critical role in modulating breast tumor cell responses to chemotherapy, either by promoting resistance mechanisms or, conversely, by enhancing chemosensitivity. The intratumoral microbiota can influence chemoresistance in breast tumor cells through several mechanisms.

#### 3.3.1. Microbial modulation of chemoresistance pathways

Certain intratumoral microbiota, notably *Fusobacterium nucleatum*, have been shown to induce chemoresistance in breast cancer cells. Mechanistically, *Fusobacterium nucleatum* can activate autophagy pathways through the TLR4/MyD88 signaling axis and specific microRNAs, leading to the protection of cancer cells from drug-induced apoptosis. In breast cancer models, colonization with *Fusobacterium nucleatum* correlates with resistance to paclitaxel and oxaliplatin, two widely used chemotherapeutic agents.^71,72^ Clearance of *Fusobacterium nucleatum* using antibiotics or targeted therapies significantly increases chemotherapy efficacy, with notable tumor volume reduction in vivo.

#### 3.3.2. Microbial enzymatic activity and drug metabolism

Intratumoral microbiota may also influence the pharmacokinetics of chemotherapeutic agents through enzymatic activities. For instance, microbial β-glucuronidase can metabolize and inactivate certain chemotherapeutic drugs, reducing their bioavailability within the tumor microenvironment. Inhibition of this enzyme activity has been associated with improved drug efficacy by maintaining higher active drug concentrations locally.^72^

#### 3.3.3. Microbiota-induced modulation of the tumor microenvironment

Chemotherapy-induced shifts in microbial composition can affect local immune modulation and inflammation, which in turn influence the response to the drug. Paclitaxel treatment has been shown to cause overgrowth of Clostridium species, leading to increased production of deoxycholic acid (DCA). Elevated DCA interacts with bile acid receptors such as Takeda G protein-coupled receptor 5 (TGR5) in the dorsal root ganglia, contributing to neuropathic pain, a common chemotherapy side effect, thus indirectly impacting patient tolerance and treatment adherence.^73^ Future research should focus on identifying specific microbial species involved in chemoresistance and exploring how modulating the microbiota could improve treatment outcomes.

### 3.4. The role of intratumoral microbiota and epigenetic modification in breast cancer

Microorganisms produce metabolites such as SCFAs, folate, and other cofactors that serve as substrates or inhibitors of epigenetic enzymes, including DNA methyltransferases (DNMTs), histone acetyltransferases (HATs), and histone deacetylases (HDACs).^74–78^ For instance, butyrate and propionate can inhibit HDACs, leading to altered histone acetylation and transcriptional changes in colorectal cancer cells.^79^ In cancer, miRNA has been proven to be a key mediator of host microbiome interactions, and bidirectional interactions between microbiome and miRNA have been observed during carcinogenesis.^80–82^ Patients with a large amount of tissue *Fusobacterium* DNA and miR21 have a higher risk of poor prognosis. Compared with patients with non-metastatic cancer (NonMet-BC), the expression of miR-149-5p, miR-20b-5p, and miR-342-5p increased in patients with metastatic breast cancer (Met-BC).^83^ The Met-BC group showed an increase in several pathogenic and pro-inflammatory species, including *Streptococcus epidermidis*, *Haemophilus influenzae*, *Corynebacterium aurimucosum*, and *Corynebacterium kroppenstedtii*, while the NonMet-BC group showed higher levels of probiotics such as *Parabacteroides distasonis*, *Lactobacillus iners*, *Blautia obeum*, and *Faecalibacterium prausnitzii*.^83^ These studies suggest that considering changes in miRNA expression and microbiota within tumors may contribute to precise cancer treatment.

### 3.5. Intratumoral microbiota and dysregulated oncogenic signaling pathways in breast cancer

The intratumoral microbiota is not a passive bystander but an active participant in the breast cancer microenvironment, capable of hijacking and dysregulating key cellular signaling pathways to promote oncogenesis.^57^ These microorganisms and their metabolites can directly interact with host cells, modulating pathways that control cell proliferation, survival, inflammation, and metastasis. Understanding these microbial-pathway interactions provides crucial insights into the mechanisms of breast cancer progression and reveals novel therapeutic vulnerabilities (Table 2). The intricate crosstalk between the intratumoral microbiota and host signaling pathways underscores its role as a key modulator of breast cancer biology.^51^ Targeting these specific microbial-pathway axes, for instance, using small molecule inhibitors against TLR4 or FadA, or modulating microbial enzyme activity, presents a promising frontier for developing novel combination therapies to overcome drug resistance and improve patient outcomes.

**Table 2.** Influence of intratumoral microbiota on oncogenic signaling pathways in breast cancer.

Microorganism/Metabolite	Signaling Pathway	Biological Effects of Breast Cancer	Ref
*Fusobacterium nucleatum*	E-cadherin/β-Catenin	Promotes DNA damage, cell proliferation	^54^
*Fusobacterium nucleatum*	TLR4/NF-κB	Induces immunosuppression, recruits MDSCs, and Tregs	^51^
HPV E6/E7 proteins	p53/Rb	Inhibits tumor suppressor pathways, promotes proliferation	^59^
SCFAs	HDAC inhibition	Alters histone acetylation, modulates gene expression	^55,84^
LPS	TLR4/MyD88	Activates autophagy, induces chemoresistance	^71^
*Candida albicans*	GPR81/VEGF	Promotes angiogenesis, lactic acid-mediated immunosuppression	^68,69^
β-glucuronidase	Estrogen signaling	Releases free estradiol, promotes ER+ breast cancer growth	^70^
TMAO	PERK/ER stress	Induces tumor cell death, enhances CD8^+^ T cell immunity	^85^

## 4. Potential clinical application of intratumoral microbiota in breast cancer

### 4.1. Intratumoral microbiota as potential biomarkers for diagnosis and prognosis of breast cancer

**Figure 4.**
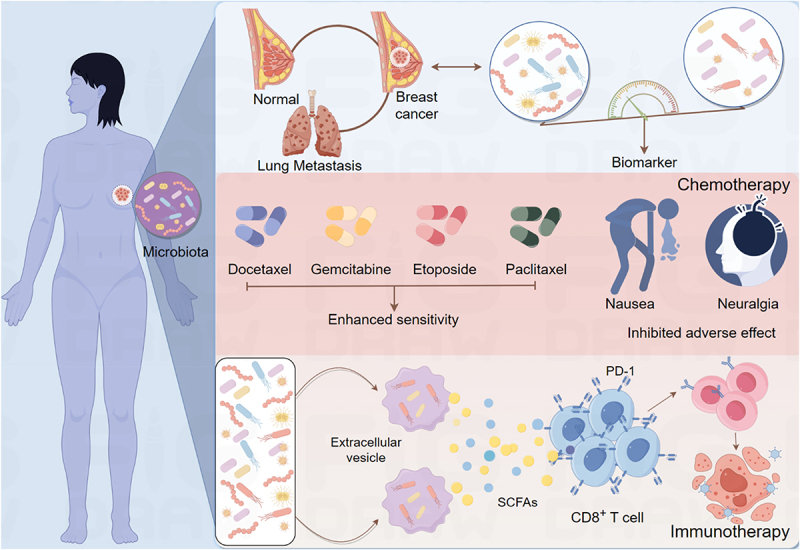
Potential clinical application of intratumoral microbiota in breast cancer therapy. The microbiome characteristics of breast tumors and matched normal tissues are very different, which can be used as potential biomarkers to distinguish tumor and non-tumor tissues. Intratumoral microbiota and their metabolites in tumors can enhance the sensitivity of breast cancer to chemotherapy drugs, such as paclitaxel, gemcitabine, and docetaxel, and can also alleviate the side effects of chemotherapy drugs, such as nausea, vomiting, and headache. Nanoparticles coated with extracellular vesicles transform the bacteria in tumors into immune enhancers to enhance the efficacy of immunotherapy in breast cancer. Made by Figdraw.

The distribution of microbial communities within tumors shows promise as a new biomarker for predicting tumor progression. When specific bacteria are enriched in tumor tissues and others in non-tumor tissues, this difference can serve as a potential biomarker to distinguish between tumor and non-tumor tissues.^86^ The microbiome characteristics of breast tumors and matched normal tissues are very different, indicating that the classification strategy using bacterial biomarkers is effective. The predictive model developed by researchers confirmed the ability to identify malignant breast tissue from bacterial features, with an accuracy of 84.78% and an AUC of 0.888.^8^ In addition, the study also found that the microbial composition in tumor tissue was closely related to tumor staging and prognosis. For example, *Fusobacterium nucleatum* is rare in primary breast cancer, but it is significantly enriched in lung metastasis.^61^ Researchers have also developed a less invasive form of biopsy core needle biopsy (CNBS). The V3 region has the highest amount of information on the microbiota of breast tissue, accounting for 45% of all readings.^87,88^ *Ralstonia* (Proteus) is more abundant in normal tissues near breast tumors and malignant tumors (Figure 4).

Previous studies have confirmed that noninvasive screening and early diagnosis of breast cancer can be achieved by detecting microbial markers in the blood.^89^ Moreover, the diversity of intratumoral microbiota is closely related to the survival rate and progression-free survival (PFS) rate of patients. These studies show that intratumoral microbiota have potential value in improving the accuracy and convenience of prognosis screening for breast cancer patients.

The microbial composition in the tumor was correlated with the prognosis of patients. Studies have shown that intratumoral microbiota, such as *Acidobacteria*, *Succinivibrionales*, *Lachnoclostridium*, and *Pseudogulbenkiania*, were related to the prognosis, tumor infiltrating immune cell abundance, and immunotherapy effect of breast cancer. Therefore, certain intratumoral microbiota may be new targets for regulating immunotherapy of breast cancer.^90^ Additionally, positive *Candida albicans* is associated with poor prognosis in breast cancer patients, and patients with positive *Candida albicans* have a significantly shortened PFS.^91^ Therefore, microbiological detection may provide an important reference for the prognosis of breast cancer. Microbial markers in blood are of particular concern. Studies have shown that the DNA level of *Fusobacterium nucleatum* in plasma can serve as an effective index for the early diagnosis of breast cancer. In one study, using the DNA detection of this bacterium in plasma, the AUC of patients with breast cancer stage I reached 0.87, which was better than the traditional tumor markers, providing a new method for early noninvasive screening of breast cancer.^92^ This method has the advantages of simple operation and noninvasive, and is expected to expand the scope of clinical application.

### 4.2. Targeting intratumoral microbiota can improve chemotherapy efficacy and alleviate side effects

Chemotherapy remains a primary treatment for breast cancer, yet its side effects and drug resistance pose significant challenges. Research indicates that chemotherapy can alter the intratumoral microbiota, such as increasing *Pseudomonas spp*. in breast cancer patients. Primary breast tumors in patients with distant metastasis showed increased tumor abundance of *Brevundimonas* and *Staphylococcus*.^61^ Some bacteria in the intratumoral microbiota, such as *Fusobacterium nucleatum*, can induce drug resistance, while some probiotics can alleviate chemotherapy side effects and enhance drug sensitivity. Antibiotics can affect the proliferation of breast cancer cells in a dose-dependent manner and regulate doxorubicin-mediated cell death.^93^ Studies have shown that intratumoral microorganisms and their metabolites can enhance the sensitivity of breast cancer to chemotherapy drugs, such as paclitaxel, gemcitabine, and docetaxel. One study found that the combination of ampicillin and paclitaxel improved the efficacy of chemotherapy.^94^ By targeting TLR4, MyD88, and specific microRNAs, *Fusobacterium nucleatum* controls oxaliplatin resistance by activating the autophagy pathway.^77^ The presence of *Fusobacterium nucleatum* is related to the drug resistance of paclitaxel. The study found that the clearance of *Fusobacterium nucleatum* could significantly improve the efficacy of paclitaxel. In the mouse model, the tumor volume reduction rate increased by 4.2 times. This mechanism may be related to the inhibition of microbial β-glucuronidase activity, thereby reducing the decomposition of drugs. In the animal model of breast cancer, the colonization of *Fusobacterium nucleatum* is associated with the reduced efficacy of chemotherapy, which is achieved by activating autophagy-related pathways in cancer cells.^72^ In addition, paclitaxel causes excessive growth of *Clostridium spp*., and the level of DCA is highly elevated. DCA increases the serum level of CCL5/CCR5 in the dorsal root ganglion through bile acid receptor TGR5, resulting in neuronal hyperexcitation and neuropathic pain.^73^ A single-center randomized clinical trial found that probiotics supplementation during docetaxel-based chemotherapy may help reduce body weight, body fat percentage, increase plasma low-density lipoprotein, and minimize metabolic changes and intestinal flora imbalance.^95^ Another randomized controlled study found that synbiotics supplementation during chemotherapy in breast cancer patients can effectively reduce the severity of fatigue and abnormal defecation, and reduce the incidence of anorexia and nausea/vomiting.^96^

### 4.3. Intratumoral microbiota can enhance immunotherapy efficacy

At present, programmed death protein 1 (PD-1)/programmed death ligand 1 (PD-L1) is widely used in immunotherapy of breast cancer. But responses are limited, and drug resistance often develops.^97^ Due to the relatively hypoxic environment inside the tumor, the activity of immune cells is inhibited, and some specific facultative anaerobes are more likely to colonize the tumor site and recruit more immune cells through their immunogenicity.^98^ The presence of intratumoral microbiota has a synergistic effect with immunotherapy, which may increase the heterogeneity of tumors and enhance the immune response. Unlike chemotherapy drugs that directly kill cancer cells, immunotherapy indirectly fights cancer cells through the patient’s immune system. Oral *Bifidobacterium* combined with anti-PD-L1 antibody therapy almost completely inhibited tumor growth by enhancing CD8^+^T cell activation.^99^ Shi et al. found that *Bifidobacterium* can accumulate in tumor tissue, promote the stimulator of interferon genes (STING) pathway, and promote CD47-based immunotherapy. Therefore, specific bacterial intervention can reverse resistance to immunotherapy.^100^ Nanoparticles coated with bacterial-derived outer membrane vesicles (OMVs) transform *Fusobacterium nucleatum* into immune enhancers, release pathogen-associated molecular patterns (PAMPs), and enhance the efficacy of immunochemical kinetic therapy in TNBC.^101^ Encapsulation of bacterial-derived extracellular vesicles (BEVS) in a nano “cloak” can increase immunogenicity and promote DC maturation by activating the cyclic GMP-AMP synthase (cGAS)- STING pathway. This method, combined with an anti-PD-L1 antibody, can trigger a strong immune response and synergistically inhibit tumor progression and lung metastasis.^102^ Therefore, vesicles derived from bacteria can effectively improve the immunosuppressive state of TME and represent a potential treatment.

## 5. The future direction of intratumoral microbiota in the treatment of breast cancer

### 5.1. Directly target intratumoral microbiota

**Figure 5.**
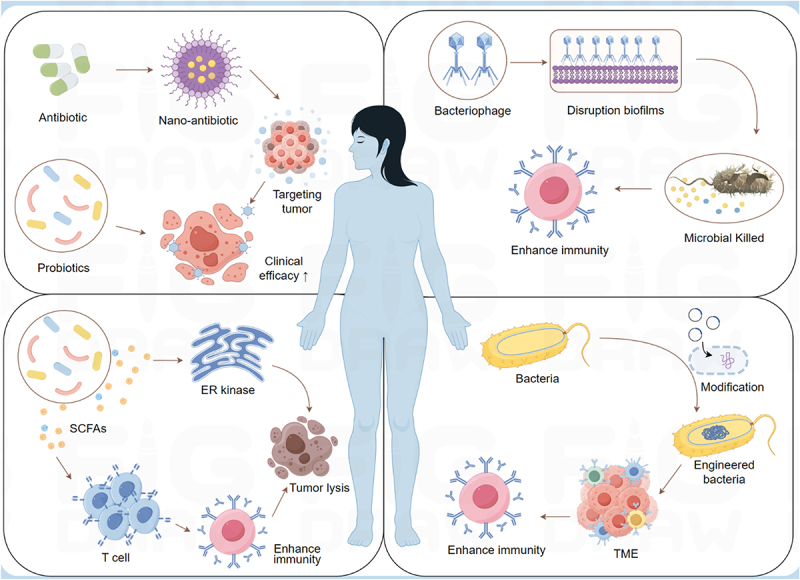
Future direction of intratumoral microbiota in breast cancer therapy. Designing nano-antibiotics can target intratumoral microbiota and develop highly targeted “precision probiotics” to improve clinical drug efficacy and achieve effective treatment and prevention of breast cancer. Bacteriophages can be modified into programmable bacterial killers, precisely targeting and eliminating harmful intratumoral microbiota, and attracting anti-tumor immune cells to attack sites. Specific microbial metabolites can activate endoplasmic reticulum stress kinase (ER kinase) PERK to induce tumor cell death, thereby enhancing breast cancer T cell-mediated anti-tumor immunity. The modified engineered bacteria can be used as an excellent drug delivery system to accurately deliver drugs to tumor lesions, regulate the tumor microenvironment, and improve the efficacy of immunotherapy. Made by Figdraw.

At present, how to improve the therapeutic effect of breast cancer by microbial therapy or adjuvant therapy has become a hot spot in clinical transformation. The use of antibiotics to remove the microbiota has been applied to prevent gastric cancer by targeted removal of *Helicobacter pylori*.^103^ Some researchers have constructed a bacteria-derived OMV-coated nanoplatform to accurately target tumor tissues. The prepared nanoparticles can effectively induce the death of breast cancer cells, thereby enhancing immunogenicity. At the same time, the tumor microorganism *Fusobacterium nucleatum* was killed by metronidazole in OMV, which significantly improved the anti-tumor immune response and achieved the effective treatment and metastasis prevention of TNBC.^101^ The development of highly targeted “Precision Probiotics” formulas and bacterial supplements may play a role in the prevention of cancer. However, most antibiotics have no different impact on the patient’s microbiota; the use of antibiotics to eliminate the microbiota in tumors is still limited, which may lead to adverse reactions and the emergence of drug-resistant strains. Targeted elimination of the tumor microbiota that promotes cancer and has minimal impact on the body is an active research field (Figure 5).

### 5.2. Phage therapy

Bacteriophages are viruses that infect prokaryotes, which can “adsorb” to host bacteria, interact with bacterial receptors through external structures, and retain the capsid outside the bacteria when their genetic material enters the bacteria.^104^ The excellent antibacterial ability and easy editing of bacteriophage make it a promising therapeutic vector targeting the tumor microbiota. Oral phages may avoid systemic immunity and directly target the disease-related microbial flora in tumors, but have little effect on other microorganisms.^105^ Therapeutic bacteriophages carrying drugs can directly target the microbiota in the tumor and release drugs in the tumor microenvironment. When the phage migrates to the tumor site where Fusobacterium nucleatum aggregates, the designed *Fusobacterium*-specific phage can carry irinotecan nanoparticles, which can be released in the tumor microenvironment, significantly improving the efficiency of first-line chemotherapy.^106^ In addition, the phage can also carry doxorubicin (DOX), a widely used chemotherapy drug, and promote drug release through the DKF motif recognized by cathepsin B, which is a lysosomal protease.^107,108^

### 5.3. Engineering bacteria

With the rapid development of nanomaterial technology, engineered bacteria have emerged by modifying bacteria to meet special needs.^109^ Engineered bacteria can be used as an excellent drug delivery system to accurately deliver drugs to tumor lesions. Genetically engineered Salmonella typhimurium expresses hemolysin e, which can effectively target tumor sites and cause cell lysis, thereby inhibiting tumor growth.^110^ Another study found that *Salmonella typhimurium* was modified to express the pro-apoptotic cytokine FasL, showing strong anti-tumor activity.^111^ In addition, engineered bacteria can also modulate the tumor microenvironment to enhance immunotherapy. The engineered probiotic *E.coli* Nissle 1917 strain can colonize tumors, increase the concentration of L-arginine in tumors, increase the number of infiltrating T cells, and has a significant synergistic effect with PD-L1 blocking antibody in tumor clearance.^112^ Although engineered bacteria have been used in cancer treatment. Security cannot be ignored.^113^ Further tests are needed in animal models and clinical practice. In a word, the development and application of engineered bacteria are of great significance to the development of cancer treatment.

### 5.4. Regulation of microbial metabolism

With the in-depth understanding of the role of microorganisms in the tumor microenvironment of breast cancer, intervention strategies based on microbial metabolism have gradually become a potential adjuvant therapy. These strategies aim to regulate microbial composition or its metabolites to improve the immune environment, inhibit tumor growth, or enhance the effectiveness of existing treatments.^114^ Clostridiales, and the related metabolic trimethylamine N-oxide (TMAO), induce tumor cell death by activating endoplasmic reticulum stress kinase PERK, thereby enhancing the anti-tumor immunity mediated by CD8^+^T cells in TNBC.^85^ At present, some clinical trials try to combine microbial regulation (probiotics, prebiotics) with traditional treatment, such as chemotherapy, immunotherapy. A randomized controlled trial (RCT) found that supplementation of lactic acid bacteria such as *Lactobacillus acidophilus* and *Bifidobacterium longum* could improve the balance of intestinal flora, reduce treatment-related gastrointestinal side effects, and regulate immune indexes.^115^ In addition, SCFAs produced by probiotics have been confirmed in some studies to enhance the immune response and potentially improve the treatment response of breast cancer.^116^ Dietary intervention, such as increasing inulin and prebiotics, can promote the reproduction of *Bifidobacterium*, increase butyrate, and inhibit the growth of breast cancer. Inulin dietary fiber can promote the proliferation of *Bifidobacterium* and increase the level of butyrate, thereby inhibiting the activity of histone deacetylase (HDAC).^84^

In summary, several clinical trials have been initiated to evaluate the potential of microbiome-based interventions in breast cancer treatment.^95,117–122^ This includes the effects of broad-spectrum antibiotics on chemotherapy and immunotherapy, the enhancing effect of probiotic supplements on immune checkpoint inhibitor response, the selective replication and anti-tumor immune stimulation of genetically engineered bacteria, such as oncolytic viruses, and the role of dietary regulation and metabolite supplementation in reshaping the microbiome and enhancing anti-cancer immunity. In addition, microbial community characteristics are also being tested in clinical trials as predictive biomarkers for treatment response and prognosis (Table 3). Although the relevant strategies are still in the experimental stage, it is expected that microbiome analysis will be incorporated into clinical workflows in the future to guide treatment decisions.

**Table 3.** The clinical trials of breast cancer diagnosis and treatment by intratumoral microbiota.

Microbe-based therapy	Status	Clinical Studies	NCT number	Ref
Intratumoral microbiota	Not recruiting	Breast cancer and its relationship with the microbiota	NCT03885648	117
Intratumorally injected Clostridium novyi-NT	Completed	This first-in-human study enrolled patients with injectable, treatment-refractory solid tumors to receive a single intratumoral injection of C. novyi-NT across 6 dose cohorts.	NCT01924689	118
Talimogene laherparepvec (T-VEC)	Completed	T-VEC can enhance TNBC responses to neoadjuvant chemotherapy (NAC).	NCT02779855	119
Oncolytic virus V937	Completed	Oncolytic virus V937 showed activity and safety with intratumoral administration.	NCT02043665	120
Intratumoral microbiota	Completed	Intratumoral microbiota serve as a strong and specific predictor of the response of patients with early-stage TNBC to Neoadjuvant immunotherapy combined with chemotherapy	A retrospective study	121
Intratumoral microbiota	Completed	Intratumoral microbiota-aided radiomics models serve as a powerful and noninvasive tool for predicting neoadjuvant chemoimmunotherapy response in early-stage TNBC patients.	A retrospective study	122
Probiotics (*Bifidobacterium longum*, *Lactobacillus acidophilus*, and *Enterococcus faecalis*)	Completed	Probiotics supplement during docetaxel-based chemotherapy for breast cancer treatment may help to minimize the metabolic changes and gut dysbiosis.	ChiCTR-INQ-17014181	95

## 6. Future outlook and conclusion

The role of intratumoral microbiota in the breast cancer microenvironment is gaining recognition, offering new avenues for early diagnosis, personalized treatment, and efficacy monitoring. Distinct microbial signatures exist in intratumoral microbiota between malignant and benign tissues, as well as across different cancer molecular subtypes. These microbial fingerprints can be accurately identified through high-resolution sequencing and spatial analysis, showing potential as noninvasive biomarkers for early cancer detection and risk stratification. Integrating microbiome data with conventional imaging and molecular diagnosis enhances the sensitivity and specificity of breast cancer diagnosis.^122^ A multi-parameter diagnostic algorithm based on microbial characteristics can distinguish between invasive and indolent tumors, advancing personalized monitoring and treatment. Dynamic changes in intratumoral microbiota composition during treatment may serve as early indicators for efficacy evaluation and drug resistance warning, facilitating real-time treatment plan adjustments.^121^

However, significant challenges remain. Firstly, technical bottlenecks limit the in-depth study of the microbiome.^123^ The abundance of microorganisms in the sample is very low, and it is easy to become contaminated, which leads to the lack of sensitivity and specificity of detection. Although high-throughput sequencing and metagenomic technology have made great progress, it is still necessary to develop more sensitive detection platforms and establish standardized sampling, storage, and analysis processes to ensure data comparability and reliability. In addition, the combination of spatial omics and single-cell technology will help to reveal the spatial distribution and dynamic changes of microorganisms in the tumor microenvironment.^124^ Secondly, the precise molecular and cellular mechanisms by which the intratumoral microbiota influence tumorigenesis, immune modulation, and therapy resistance remain incompletely understood. Thirdly, clinical translation also faces numerous obstacles. The highly individualized nature of the microbiome, influenced by genetics, environment, lifestyle, and antibiotic exposure, complicates biomarker generalization and therapeutic design.^125^ The influence of diet, antibiotics, and host genetics on the tumor microbiome’s composition and function warrants comprehensive investigation. Although engineered bacteria and bacteriophages show promise, their safety, stability, and immunogenicity require further evaluation. Safely controlling the microbiome remains a critical challenge.

Future direction will focus on multi-omics integration. Combining metagenomics, transcriptomics, proteomics, and spatial omics can create a comprehensive map of the microbe-host-tumor landscape. This will deepen our understanding of microbial mechanisms in breast cancer development, such as exploring the mechanisms of intratumoral microbiota, complement cascade, and epigenetic modifications within breast cancer, and provide a basis for precise interventions.^126^ Intelligent probiotics designed to respond to tumor microenvironment signals and release anti-tumor metabolites represent a new generation of “Biological Drugs,” offering precision treatment possibilities. Fecal microbiota transplantation (FMT) aims to improve the microenvironment and enhance immunity in breast cancer patients. While preclinical and early clinical trials show potential, safety, and long-term effects require further validation. Integrating big data and artificial intelligence will enable microbiome-based personalized analysis and tailored microbial regulation strategies. For instance, selecting specific probiotics or dietary interventions based on individual microbiome profiles can optimize intervention efficiency.

In conclusion, the research on intratumoral microbiota in breast cancer is in a nascent but rapidly evolving stage. With the continuous breakthrough of new technologies and the in-depth integration of multiple disciplines, microbiomics is expected to become an important tool for early diagnosis, treatment optimization, and efficacy monitoring of breast cancer. This will provide patients with more precise and safer treatment options and improve prognosis.

## Data Availability

Not Applicable.
